# Spontaneous mutational patterns and novel mutations for bedaquiline and clofazimine resistance in *Mycobacterium tuberculosis*

**DOI:** 10.1128/spectrum.00090-23

**Published:** 2023-08-30

**Authors:** Jin Shi, Yuanyuan Liu, Tuoya Wu, Lu Li, Shujing Han, Xiao Peng, Yuanyuan Shang, Yongli Guo, Yu Pang, Mengqiu Gao, Jie Lu

**Affiliations:** 1 Department of Tuberculosis, Beijing Chest Hospital, Capital Medical University/Beijing Tuberculosis & Thoracic Tumor Research Institute, Beijing, China; 2 Beijing Key Laboratory for Pediatric Diseases of Otolaryngology, Head and Neck Surgery, Beijing Pediatric Research Institute, Beijing Children’s Hospital, Capital Medical University, National Center for Children’s Health, Beijing, China; 3 Department of Tuberculosis Diseases, Tongliao Infectious Disease Hospital, Tongliao, Inner Mongolia, China; 4 Department of Bacteriology and Immunology, Beijing Chest Hospital, Capital Medical University/Beijing Tuberculosis & Thoracic Tumor Research Institute, Beijing, China; AP-HP, Paris, France

**Keywords:** *Mycobacterium tuberculosis*, bedaquiline, clofazimine, resistance

## Abstract

The 2022 World Health Organization guidelines recommend use of two core anti-tuberculosis (TB) drugs, bedaquiline (BDQ) and clofazimine (CFZ), for treatment of drug-resistant (DR)-TB. However, several mutated *Mycobacterium tuberculosis* (MTB) genes, conferring BDQ and CFZ resistance, have been reported that predominantly arose from sporadic mutations that have not been comprehensively characterized. Herein, MTB clinical isolates collected from drug-susceptible (DS)-, multidrug-resistant (MDR)-, and extensively drug-resistant (XDR)-TB patients were cultured *in vitro* with BDQ or CFZ to generate progeny strains with resistance to these drugs. Progeny strains exposed to CFZ exhibited increased CFZ minimum inhibitory concentrations (MICs) that exceeded MIC increases of BDQ-exposed progeny strains. Notably, *mmpR* and *pepQ* mutations accounted for 83% and 17% of BDQ-induced spontaneous gene mutations, respectively, and 86% and 14% of CFZ-induced spontaneous gene mutations, respectively. Analyses of predicted mutation-induced changes in amino acid sequences and structures of MmpR and PepQ mutants revealed several point mutations affected sequence conversation and functionality as an underlying mechanism for observed acquired BDQ/CFZ resistance. Moreover, our results revealed differences in patterns of BDQ- and CFZ-induced acquired spontaneous mutations that may enhance our understanding of MTB BDQ/CFZ-resistance mechanisms.

**IMPORTANCE:**

This study of MTB drug resistance mechanisms revealed patterns of spontaneous MTB mutations associated with acquired BDQ and CFZ resistance that arose after clinical MTB isolates were cultured *in vitro* with BDQ or CFZ. Results of protein sequence and structural analyses provided insights into potential mechanisms underlying associations between MTB gene mutations and DR phenotypes. Taken together, these results revealed differences in acquired BDQ and CFZ resistance mechanisms as a new perspective that may enhance our understanding of BDQ/CFZ resistance mechanisms and facilitate the development of new methods for detecting MTB drug resistance genes.

## INTRODUCTION

Tuberculosis (TB), an infectious disease caused by *Mycobacterium tuberculosis* (MTB), remains a major public health issue and one of the leading causes of human deaths worldwide (1). Notably, the emergence of drug-resistant TB (DR-TB), especially multidrug-resistant TB (MDR-TB), has greatly hindered global TB control efforts, as have the following factors: low DR-TB treatment success rates, long DR-TB treatment durations, and interrupted anti-TB treatment administration during the COVID-19 epidemic (2 – 4). Therefore, new anti-TB drugs are urgently needed to stop the spread of DR-TB.

Bedaquiline (BDQ) and clofazimine (CFZ) are core anti-TB drugs recommended by 2022 World Health Organization (WHO) guidelines for the treatment of DR-TB cases, with both drugs predominantly used to treat multidrug-resistant/rifampicin-resistant TB (MDR/RR-TB) (5). BDQ is a member of one of only two new anti-TB drug classes that have been approved for clinical use in over half a century. Notably, results of studies conducted in South Africa have demonstrated that administration of the 6-mo BPaL (bedaquiline + pretomanid + linezolid) regimen to extensively drug-resistant TB (XDR-TB) and MDR-TB patients with histories of poor treatment outcomes achieved high treatment success rates of about 90% (6). Moreover, results of other clinical studies have shown that successful treatment of DR-TB cases with BDQ-containing regimens could be completed in shorter periods of time than conventional treatments, while incorporation of BDQ in all-oral treatment regimens simplified anti-TB treatment and enhanced patient compliance (6). Meanwhile, CFZ, a key drug used to treat leprosy patients (7), has been shown to significantly inhibit MTB growth both *in vitro* and *in vivo* (8, 9). In fact, results of multiple clinical studies have demonstrated high success rates of CFZ-containing chemotherapy regimens that ranged from approximately 84% to 89% (10 – 13). Furthermore, results of other studies have shown that treatment of MDR-TB patients with such regimens could achieve successful outcomes when administered for shorter periods of time than are required for completion of conventional anti-MDR-TB treatments (14, 15). Based on the abovementioned results, BDQ and CFZ are currently under evaluation in several clinical trials, including the ongoing Phase III BEAT-TB clinical trial.

Nevertheless, in spite of improved TB cure rates of BDQ- and CFZ-containing anti-TB regimens, in recent years, TB cases with resistance to BDQ and CFZ and cross-resistance between the two drugs have been reported. Consequently, investigations of mechanisms underlying resistance to BDQ and CFZ have been conducted that have led to discoveries of several genes associated with phenotypic resistance to these drugs. For example, mutations of *atpE* (*Rv1305*), *mmpR* (*Rv0678*), and *pepQ* (*Rv2535c*) have been associated with phenotypic resistance to BDQ, while mutations of the latter two genes have been implicated in cross-resistance to CFZ and BDQ (16 – 18). However, due to the fact that these drugs have only been clinically used for a few years, few clinical isolates with resistance to these drugs have been reported. More recently, these isolates have been shown to mainly harbor sporadic gene mutations that have yet been comprehensively characterized as an explanation for why high-confidence BDQ or CFZ resistance variants have not yet been announced by WHO (19). Nonetheless, the emergence of BDQ and CFZ resistance-inducing mutations has led to declining effectiveness of these drugs (20 – 25). To address this issue, researchers are urgently studying mechanisms underlying MTB resistance to BDQ and CFZ toward developing rapid DR-TB early detection methods and new drugs to prevent DR-TB emergence and transmission.

In this study, clinical MTB isolates were cultured with BDQ or CFZ *in vitro* to generate MTB progeny strains with acquired resistance to these drugs. Thereafter, drug-treated progeny strains were analyzed to identify drug resistance-associated gene mutations, then gene mutation-induced changes in protein sequences and structures were predicted in order to identify mutations associated with observed drug resistance. The results of this study provide new clues to guide future research efforts toward development of new methods for detecting MTB anti-TB drug resistance genes.

## MATERIALS AND METHODS

### Clinical strains

The strains in this study were classified as drug-susceptible (DS)-, MDR-, and XDR-TB strains according to the pre-2021 WHO definition (26). Ultimately, 15 DS, 15 MDR and 15 XDR MTB strains derived from clinical isolates (collected from March to September 2016) were obtained from the Biobank of Beijing Chest Hospital in China. Each MTB strain was isolated from a unique TB patient. Phenotypic resistance to first- and second-line drugs was determined using the absolute concentration method, as described in the aforementioned WHO recommendations (27).

### Minimal inhibitory concentration determinations

The resazurin microtiter assay (REMA) was conducted to determine BDQ and CFZ minimal inhibitory concentrations (MICs) for the abovementioned clinical MTB strains (28). Concentrations of initial stock solutions of CFZ and BDQ (dissolved in dimethyl sulfoxide solvent) were both 6,400 µg/mL. To prepare each MTB inoculum, a cell suspension of each isolate with turbidity equivalent to that of a McFarland 1.0 standard was diluted 1:20 in Middlebrook 7H9 broth containing 10% oleic acid-albumin-dextrose-catalase (OADC). Serial twofold dilutions of BDQ or CFZ in 100 µL volumes of 7H9 broth were directly prepared in wells of 96-well plates in concentrations ranging from 0.016 μg/mL to 16 μg/mL. Next, 100 µL of the diluted inoculum was added to each well, then plates were incubated at 37°C for 7 days. After incubation, 40 µL of 0.01% fresh resazurin solution was pipetted into each well, then results were read after an additional 24 h incubation at 37°C. The MIC for each isolate was determined as the lowest drug concentration that prevented a color change from blue to pink, with the MTB H37Rv strain (ATCC 27294) serving as a control for assay performance that was included in all assay runs. Clinical isolates with MICs below the tentative critical drug concentration (BDQ: 0.125 µg/mL, CFZ: 1 µg/mL) were scored as DS results (29, 30); isolates with MICs that were equal to the tentative critical concentration fell within the area of technical uncertainty, meaning that sensitivity or resistance could not be determined (31); strains with MICs that were greater than the abovementioned tentative critical concentrations were scored as DR strains.

### Isolation of spontaneous mutants

We further adapted the method of Ismail et al. to generate spontaneous mutants of clinical strains (21). Briefly, a fresh culture of each clinical strain was harvested, then cultured in order to generate an inoculum with turbidity equivalent to that of a McFarland 1.0 standard. Next, 100 µL of the inoculum was inoculated onto two 7H10 agar plates, of which one plate contained BDQ and the other contained CFZ. For BDQ, 0.25 µg/mL was selected as the tentative critical concentration for 7H10 agar screening, as based on results of previous studies (32, 33). Due to the fact that the 7H10/7H11 agar method has not been reported as a method for determining CFZ critical concentration, 1 µg/mL was chosen as the tentative CFZ critical concentration (as based on our REMA results). Thereafter, two concentrations (0.5× and 1× of the tentative critical concentration) of each drug (BDQ: 0.125 µg/mL and 0.25 µg/mL; CFZ: 0.5 µg/mL and 1 µg/mL) were incorporated in solid medium within culture plates, then a total of 12 plates was inoculated (three plates for each of the two concentrations for each drug). Four weeks after inoculation, progeny strains growing on drug-containing plates were harvested, then MICs for all strains were determined. Genomic DNA was isolated from each strain, then subjected to DNA sequence-based analysis.

### Amplification and sequencing of resistant genes conferring BDQ and/or CFZ resistance

Genomic DNA extraction and sequencing were performed using DNA sequencing and amplification primers as reported previously (24). Coding regions of *atpE*, *mmpR,* and *pepQ* were amplified and sequenced for BDQ-treated progeny strains and corresponding parent strains, while coding regions of *mmpR*, *pepQ*, and *Rv1979c* were amplified and sequenced for CFZ-treated progeny strains and corresponding parent strains. Each PCR was performed in a final volume of 50 µL containing 5 µL PCR buffer, 2 mM MgCl_2_, 200 µM of each dNTP, 0.2 µM of each primer set, and 1 U HotStar Taq polymerase (Qiagen). Amplified products were sent to the Tsingke Company (Beijing, China) for DNA sequence analysis. DNA sequences were aligned to corresponding sequences of the standard laboratory MTB strain H37Rv using BioEdit Sequence Alignment Editor version 7.1 (https://bioedit.software.informer.com/7.1/).

### Spoligotyping

Genotyping of MTB was performed using a MeltPro *Mycobacterium tuberculosis* McSpoligotyping Kit (Xiamen Zeesan Biotech, Xiamen, P. R. China) as described in previous studies (34). Each amplification reaction was prepared in a final volume of 25 µL that contained 19.75 µL of McSpoligotyping PCR Mix (A/B/C), 0.25 µL of McSpoligotyping enzyme, and 5 µL of DNA. Thermal cycling was conducted using a Zeesan SLAN96 real-time fluorescent PCR instrument under PCR cycling conditions as specified by the instructions provided with the kit. The melt curve analysis procedure was performed under the following conditions: 95°C for 3 min, then 35°C for 1 min followed by a temperature increase from 35°C to 90°C at a rate of 0.04°C/s to acquire fluorescence signals. The results were submitted to the SITVIT2 database (http://www.pasteur-guadeloupe.fr:8081/SITVIT2/submit.jsp) and used to assign spoligotype international types and corresponding clades to our MTB isolates.

### Analysis of protein sequences and structures

We first performed BLAST against the UniProt database (https://www.uniprot.org/blast/) to find orthologs of MmpR and PepQ. Sequence alignment was performed using ClustalO (35), then results were formatted and visualized using ESPript (36). The PROVEAN score (37), a mutation impact score based on a multiple sequence alignment of protein sequences against the non-redundant protein sequence database, was calculated for each mutation using TBvar3D (https://swissmodel.expasy.org/var3d/).

Structures of mutant proteins were modeled using SWISS-MODEL (http://swissmodel.expasy.org/), and templates were obtained from Protein Data Bank (PDB) (https://www.rcsb.org/) and AlphaFold Protein Structure Database (http://alphafold.ebi.ac.uk/). Templates used to model mutant MmpR and PepQ proteins included PDB file 4NB5 and AlphaFold file AF-I6YDN6-F1, respectively, while the PDB file 1Z9C template was used to model the MmpR-DNA complex.

Free energy changes were calculated using Eris (38) and PremPS (39) to predict impacts of point mutation-induced amino acid sequence changes on protein stability. Detailed local interaction changes were analyzed using the “Structure Monitor” module of Discovery Studio Visualizer v.4.5 (BIOVIA, Dassault Systèmes, San Diego, CA, USA).

## RESULTS

### Characterization of parent strains

Due to culture contamination or initial BDQ- or CFZ-resistance, nine strains were excluded. Of the remaining 36 strains, 30 strains with MICs that fell below tentative critical MIC values indicative of BDQ or CFZ drug resistance (10 DS, 10 MDR, and 10 XDR) were randomly selected to serve as parent strains, with MIC values of the 30 parent strains shown in Table 1. Results of spoligotyping analysis indicated that 27 (90%) of the 30 parent strains belonged to the Beijing MTB family, including all 20 MDR and XDR strains and 7 of the DS strains (70%), while 3 of the DS strains (30%) belonged to the T1 MTB family (Table S1).

**TABLE 1:** MIC distribution of parent strains

Drugs	Strains	No. of strains with different MICs (µg/mL)											Breakpoint (µg/mL)
		≤0.016	0.03	0.06	0.125	0.25	0.5	1	2	4	8	16	
BDQ	DS^*a*^	1	5	3	1	0	0	0	0	0	0	0	0.125
	MDR^*b*^	3	5	2	0	0	0	0	0	0	0	0	
	XDR^*c*^	1	3	6	0	0	0	0	0	0	0	0	
CFZ	DS	0	0	0	0	5	5	0	0	0	0	0	1
	MDR	0	0	0	1	3	6	0	0	0	0	0	
	XDR	0	0	0	1	5	4	0	0	0	0	0	

^*a*^ DS: drug susceptible.

^*b*^ MDR: multidrug resistant.

^*c*^ XDR: extensively drug resistant.

After 4 wk of culture, progeny strains treated with BDQ or CFZ were harvested and analyzed; BDQ-treated strains could only be obtained from plates with 0.125 µg/mL BDQ (0.5× of the tentative critical BDQ MIC), not from plates with 0.25 µg/mL BDQ (the tentative critical BDQ MIC). Ultimately, progeny strains were obtained from nine parent strains (30%) that included four DS strains (DS-1, DS-3, DS-6, and DS-10), two MDR strains (MDR-7 and MDR-8), and three XDR strains (XDR-1, XDR-7, and XDR-9). After CFZ treatment, 21 parent strains (70%) yielded progeny strains that grew on plates containing 0.5 µg/mL CFZ (0.5× of the tentative critical CFZ MIC), while 11 parent strains (37%) yielded progeny strains that grew on plates containing 1 µg/mL CFZ (the tentative critical CFZ MIC). The 21 parent strains that yielded progeny strains when cultured on CFZ plates included seven DS strains (DS-2 to DS-6, DS-8, and DS-9), seven MDR strains (MDR-1 to MDR-4 and MDR-7 to MDR-9), and seven XDR strains (XDR-1, XDR-3 to XDR-6, XDR-9, and XDR-10). Notably, six parent strains (DS-3, DS-6, MDR-7, MDR-8, XDR-1, and XDR-9) yielded progeny strains when cultured on both BDQ plates and CFZ plates (Table S2).

### Progeny strain drug resistance gene mutations and MICs

After BDQ exposure, 13 progeny strains were obtained, of which only one strain lacked mutations within sequenced regions of *mmpR*, *pepQ,* and *atpE* genes. Of the remaining 12 progeny strains, 10 strains (83%) possessed mutations within *mmpR* and 2 strains (17%) possessed mutations within *pepQ*, while no *atpE* mutation was detected. Analysis of these mutations (five point mutations, two frameshift mutations, and two stop codon-generating mutations) indicated that they would induce nine different types of amino acid sequence alterations (Table 2). After progeny strains were exposed to BDQ, their BDQ MICs ranged from 0.125 µg/mL to 0.25 µg/mL and exhibited four- to eightfold BDQ MIC increases relative to corresponding parental strain MICs. Interestingly, all BDQ-induced mutations led to CFZ resistance, as reflected by CFZ MICs of these strains that ranged from 2 µg/mL to 4 µg/mL. In addition, all mutant MmpR and PepQ proteins (except for MmpR G24C and G65E mutants) were associated with greater fold changes in CFZ MICs relative to parent MICs as compared to corresponding fold changes observed for BDQ MICs (Table 2).

**TABLE 2:** Mutations in BDQ resistance-conferring genes

Genes	Nucleotide change	Amino acid change	MIC (µg/mL)		No. of strains	MIC change fold^*e*^	
			BDQ	CFZ		BDQ	CFZ
*mmpR*	32delG^*a*^	G11fs^*c*^	0.125	2	2	4	16
*mmpR*	70G > T	G24C	0.25	2	1	8	8
*mmpR*	134T > G	V45G	0.125	4	1	4	16
*mmpR*	194G > A	G65E	0.25	2	1	4	4
*mmpR*	233G > C	G78A	0.25	2	2	4	8
*mmpR*	253G > T	V85P	0.125	2	1	4	8
*mmpR*	466C > T	R156^*d*^	0.25	2	2	4	8
*pepQ*	347_348insG^*b*^	D116fs	0.25	4	1	4	16
*pepQ*	735G > A	W245^*d*^	0.25	4	1	4	16

^*a*^ del: deletion.

^*b*^ ins: insertion.

^*c*^ fs: frame shift.

^*d*^ stop codon.

^*e*^ MIC change fold relative to parent MIC.

After CFZ exposure, a total of 186 progeny strains was obtained, of which approximately one-fourth (46 strains) was randomly selected for culture by taking one progeny strain colony from each plate. During the culturing of progeny strains, four strains were excluded due to contamination, yielding 42 progeny strains that were subjected to further analyses. Of these, 20 progeny strains lacked *atpE*, *pepQ,* and *mmpR* mutations, while 19 of the remaining 22 strains (86%) possessed *mmpR* mutations and 3 strains (14%) possessed *pepQ* mutations. Analysis of strains with mutations within sequenced regions of *atpE*, *pepQ,* and *mmpR* revealed a total of 21 distinct types of amino acid sequence-altering mutations that included 12 point mutations, 5 frameshift mutations, 1 insertion, and 3 stop codon-generating mutations (Table 3). For CFZ-exposed progeny strains, BDQ and MICs ranged from 0.03 µg/mL to 0.5 µg/mL and CFZ MICs ranged from 1 µg/mL to 4 µg/mL, while progeny strain MICs were 2- to 16-fold greater than corresponding parent strain MIC values.

**TABLE 3:** Mutations in CFZ resistance-conferring genes

Genes	Nucleotide change	Amino acid change	MIC (µg/mL)		No. of strains	MIC change fold	
			BDQ	CFZ		BDQ	CFZ
*mmpR*	32delG^*a*^	G11fs^*c*^	0.125	2	1	4	16
*mmpR*	109G > C	G37R	0.25	1	1	8	2
*mmpR*	116T > C	L39S	0.06	1	1	2	2
*mmpR*	119T > G	L40W	0.5	4	1	4	8
*mmpR*	124T > C	W42R	0.25	4	1	4	8
*mmpR*	134T > G	V45G	0.125	4	2	4	16
*mmpR*	151C > T	Q51^*d*^	0.125	2	1	2	4
*mmpR*	194G > A	G65E	0.25	2	1	16	4
*mmpR*	198delG	G66fs	0.5	4	1	8	16
*mmpR*	226C > T	Q76^*d*^	0.125	1	1	4	2
*mmpR*	248T > C	L83P	0.5	2	1	16	4
*mmpR*	257C > T	A86V	0.125	2	1	8	8
*mmpR*	278T > C	F93S	0.5	4	1	4	8
*mmpR*	302C > A	A101E	0.03	1	1	≥2	4
*mmpR*	412_413insG^*b*^	E138fs	0.5	4	1	8	16
*mmpR*	425T > C	L142P	0.125	2	1	4	4
*mmpR*	435_436ins GCGGATTTC ACAAAGCAG	Y145_M146 insADFTKQ	0.125	1	1	4	2
*mmpR*	436delA, 437delT	M146fs	0.25	2	1	8	8
*pepQ*	735G > A	W245^*d*^	0.125	2	1	2	8
*pepQ*	947T > G	L316R	0.25	2	1	4	8
*pepQ*	1060delA	T354fs	0.5	2	1	8	8

^*a*^ del: deletion.

^*b*^ ins: insertion.

^*c*^ fs: frame shift.

^*d*^ stop codon.

### Contributions of mutations to drug resistance

**Fig 1:**
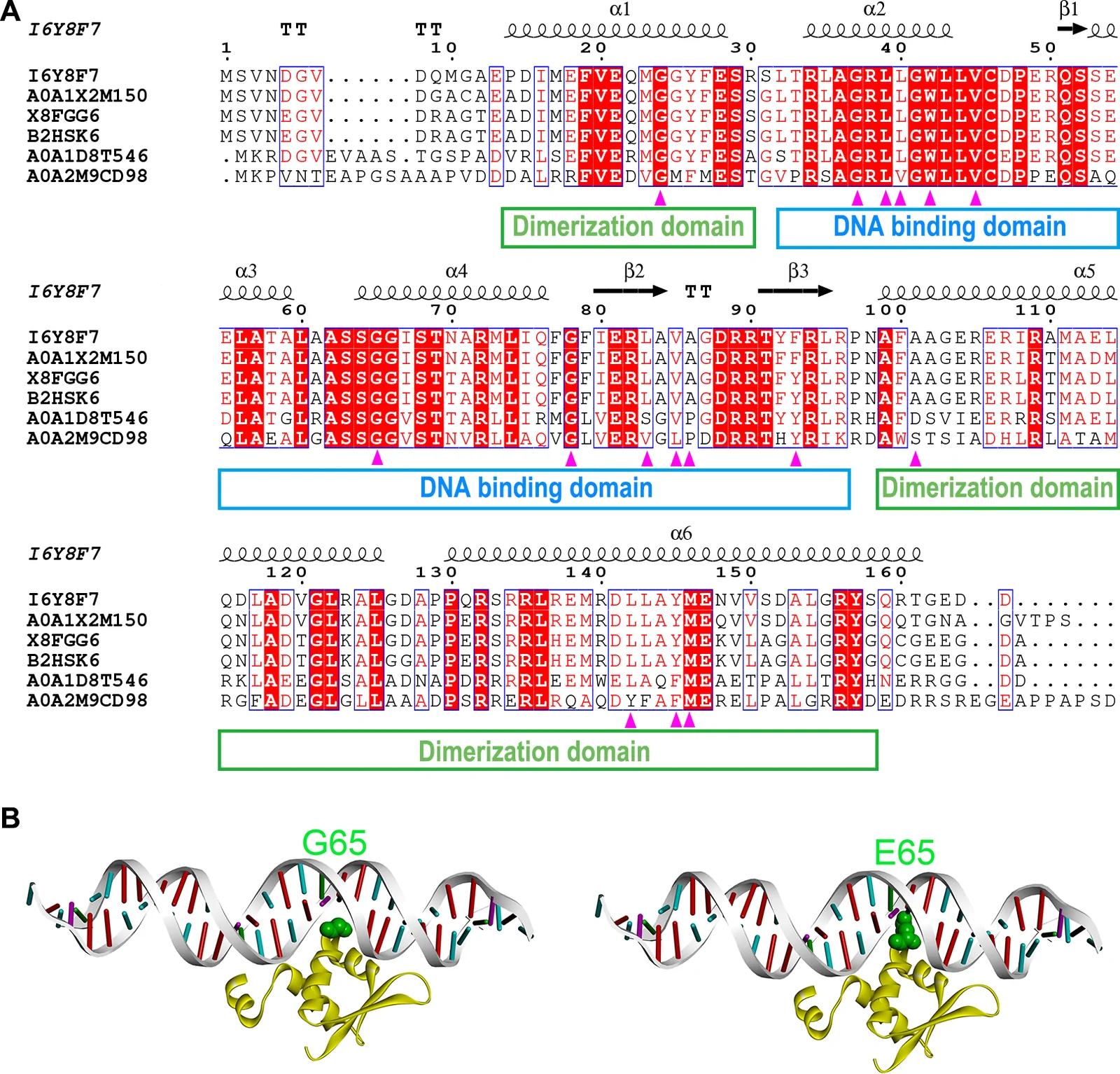
Sequence and structural analyses of MTB MmpR. (A) Multiple sequence alignments of MTB MmpR and its orthologs. According to BLAST results, the five orthologs of MmpR (UniProt entry I6Y8F7) with highest BLAST scores included a transcriptional regulator from *Mycobacterium decipiens* (UniProt entry A0A1 × 2M150), the putative regulatory protein MarR from *Mycobacterium ulcerans* (UniProt entry X8FGG6), a transcriptional regulator from *Mycobacterium marinum* (UniProt entry B2HSK6), a transcriptional regulator from *Rhodococcus* sp. WMMA185 (UniProt entry A0A1D8T546), and a MarR family protein from *Sediminihabitans luteus* (UniProt entry A0A2M9CD98). (B) Structures of wild-type MmpR (left) and MmpR G65E mutant (right) within MmpR-DNA complexes. MmpR protein (yellow) and DNA (gray) molecular structures are displayed in cartoon mode with residue 65 indicated in green, as based on the Corey–Pauling–Koltun color convention.

MmpR is a transcriptional repressor of MmpL5 and MmpS5, whereby mutations that inactivate MmpR may lead to increased MmpL5 and MmpS5 levels and elevated CFZ and BDQ MICs (18). To evaluate impacts of point mutations on MmpR function, we first performed MmpR sequence analysis, with results shown in Fig. 1A. Notably, MmpR residues affected by these mutations (G24, G37, L39, W42, V45, G65, and G78) were highly conserved, while residues 86 and 101 were not conserved. In addition, all MmpR mutation-induced amino acid substitutions (except for V85F, A86V, and A101E) yielded markedly reduced Protein Variation Effect Analyzer (PROVEAN) scores (below −2.282), which indicated that these mutations reduced protein sequence similarities between MmpR and sequences of many other functionally orthologous proteins (Table 4). Moreover, an analysis of predicted effects of point mutation-induced MmpR structural alterations on protein stability provided insights into MTB BDQ/CFZ drug resistance mechanisms. The L-shaped MmpR monomer structure includes a shorter domain (residues 32–97) that participates in DNA binding and a longer dimerization domain (residues 14–30 and 99–158). Interestingly, of the 14 BDQ/CFZ-induced MmpR mutations detected in this study, 11 mutations led to amino acid substitutions (G37R, L39S, L40W, W42R, V45G, G65E, G78A, L83P, V85F, A86V, and F93S) within the DNA-binding domain, while the other 3 mutations (G24C, A101E, and L142P) were found in the dimerization domain (Fig. 1A). Of the DNA-binding domain mutant, only the G65E amino acid substitution was predicted to directly influence DNA binding by inducing the formation of a bump that would disrupt binding between the MmpR glutamic acid side chain and the DNA molecule (Fig. 1B); the other mutation-induced amino acid sequence alterations may indirectly influence DNA binding or domain dimerization through effects on local protein structures. Meanwhile, results of detailed structural analysis suggested that mutation-induced amino acid substitutions G24C, G37R, L40W, and V85F cause structural instability by promoting formation of protein structure-disrupting bumps, while L39S, W42R, V45G, and F93S substitutions would lead to loss of hydrophobic interactions. Furthermore, substitutions of non-proline amino acids with proline residues may alter the directional orientation of the protein backbone to reduce hydrogen bonding interactions that might disrupt α-helix (L142P) and β-sheet (L83P) structures. Taken together, our predicted results indicate that all of the abovementioned mmpR amino acid changes except for A86V may lead to protein instability (Table 4) and that all MmpR point mutation-induced amino acid substitutions other than A86V and A101E may contribute to MTB BDQ and CFZ resistance (Table 4).

**TABLE 4:** Protein sequence and structure analysis of point mutations

Gene	Amino acid change	Sequence conservation	PROVEAN score^*a*^	ΔΔG (kcal/mol)^*b*^		Changes of interaction			Predicted contribution to drug resistance	MIC (µg/mL)		No. of strains
				Eris	PremPS	Local structure	DNA binding	Dimerization		BDQ	CFZ	
*mmpR*	G24C	High	−3.79	NA	0.88	+ Bump^*c*^	None	None	Resistance	0.25	2	1
*mmpR*	G37R	High	−7.03	1.13	0.98	+ Bump	None	None	Resistance	0.25	1	1
*mmpR*	L39S	High	−3.14	5.72	2.92	− Hydrophobic	None	None	Resistance	0.06	1	1
*mmpR*	L40W	Mediate	−3.54	9.31	1.26	+ Bump	None	None	Resistance	0.5	4	1
*mmpR*	W42R	High	−5.93	>10	0.82	− Hydrophobic	None	None	Resistance	0.25	4	1
*mmpR*	V45G	High	−6.17	5.77	1.53	− Hydrophobic	None	None	Resistance	0.125	4	3
*mmpR*	G65E	High	−4.5	8.39	0.03	None	+ Bump	None	Resistance	0.25	2	2
*mmpR*	G78A	High	−4.15	0.67	0.93	**None**	**None**	**None**	Resistance	0.25	2	2
*mmpR*	L83P	Mediate	−2.88	5.41	1.87	− β-Sheet	None	None	Resistance	0.5	2	1
*mmpR*	V85F	Mediate	−**0.61**	1.15	0.24	+ Bump	None	None	Resistance	0.125	2	1
*mmpR*	A86V	**Low**	−**1.42**	1.28	−**0.05**	**None**	**None**	**None**	Uncertain	0.125	2	1
*mmpR*	F93S	Mediate	−5.8	3.2	2.73	− Hydrophobic	None	None	Resistance	0.5	4	1
*mmpR*	A101E	**Low**	**0.25**	1.65	0.04	**None**	**NA**	**None**	Uncertain	0.03	1	1
*mmpR*	L142P	Mediate	−4.08	>10	1.5	− α-Helix	NA	None	Resistance	0.125	2	1
*pepQ*	L316R	High	−5.41	>10	3.01	− Hydrophobic	NA	NA	Resistance	0.25	2	1

^*a*^ A mutation impact score based on a multiple sequence alignment of the protein sequence against the non-redundant protein sequence database, a score of lower than −2.282 was considered to have a deleterious effect. The values higher than −2.282 were indicated in bold.

^*b*^ The free energy (ΔΔG) was calculated for point mutations by using two endpoint methods, namely, Eris and PremPS. ΔΔG value higher than 0 indicated that the mutation might destabilize the protein structure. The value lower than 0 was indicated in bold.

^*c*^ + Bump, introduction of unfavorable bump; − Hydrophobic, loss of hydrophobic interaction; − α-Helix, disruption of α-helix; − β-Sheet, disruption of β-sheet. Mutations without any change of interaction were indicated in bold.

The function of PepQ is still unclear. However, BLAST results revealed that the PepQ L316 residue was highly conserved. Meanwhile, the PepQ mutation that induced the L316R substitution markedly reduced the PROVEAN score to below −2.282 as an indication that this mutation reduced the similarity between the mutant MTB PepQ sequence and sequences of MTB PepQ orthologs. Moreover, predicted free energy changes (calculated using Eris and PremPS) suggested that this amino acid substitution would potentially cause structural instability of PepQ, as consistent with results of a detailed structural analysis suggesting that the PepQ L316R substitution led to loss of hydrophobic interaction(s) (Table 4). Taken together, the abovementioned sequence and structural analysis results indicate that the PepQ L316R amino acid substitution may contribute to drug resistance (Table 4).

## DISCUSSION

In the present study, we performed *in vitro* experiments to generate progeny strains with increased BDQ and/or CFZ resistance that were derived from DS, MDR, and XDR-TB clinical isolates (parent strains). Subsequent sequencing of coding regions of several genes associated with progeny strain phenotypic resistance to these drugs resulted in the identification of novel gene mutations. Results of analyses of predicted amino acid sequence and protein structural changes induced by detected BDQ/CFZ-induced *mmpR* and *pepQ* mutations revealed potential impacts of these point mutations on MTB phenotypic expression of BDQ and/or CFZ resistance.

Results of previous studies have confirmed that phenotypic cross-resistance between BDQ and CFZ can occur in clinical MTB isolates with resistance to these drugs, including MTB strains with *in vitro*-induced BDQ and CFZ resistance and strains with BDQ/CFZ resistance acquired *in vivo* during MTB infection of mice (16, 18, 20, 22, 24, 40). Similarly, cross-resistance between BDQ and CFZ was observed in this work, as evidenced by elevated MICs observed after *in vitro* exposures of progeny strains to either of the two drugs. Specifically, all mutations induced by BDQ treatment resulted in CFZ resistance, as reflected by CFZ MICs within the range of 2‒4 µg/mL. In addition, each mutation-induced mutant protein (except for G24C and G65E) exhibited a fold change in CFZ MIC (relative to that of the parent strain) that was greater than that observed for its corresponding BDQ MIC.

In this work, 1 BDQ-treated progeny strain (8%) and 20 CFZ-treated progeny strains (48%) harbored no gene mutations in sequenced regions of *mmpR*, *pepQ,* and *atpE*, as consistent with results of previous studies (41 – 43). There are several possible explanations for the lack of resistance mutations within gene sequences of isolates with phenotypic resistance to these drugs, such as the presence of undetected mutations within other drug resistance genes, as well as false-positive drug resistance results. Nevertheless, spontaneous BDQ-induced *mmpR* mutations accounted for the highest proportion of detected mutations (83%), while the remaining mutations (17%) were detected within *pepQ*. Importantly, we did not identify any mutations within *atpE*, as consistent with results of previously reported studies showing that such mutations were infrequently detected in clinical samples (23, 44). Meanwhile, spontaneous CFZ-induced mutations detected here within *mmpR* and *pepQ* accounted for 86% and 14% of detected mutations, respectively. In other studies, *in vitro* exposure of the MTB H37Rv strain to BDQ or CFZ led to the generation of progeny strains with resistance to these drugs, of which 93% (157/168) of BDQ-resistant strains and 97% (93/96) of CFZ-resistant strains harbored *mmpR* mutations (25, 45). Similarly, most gene mutations detected in clinical isolates obtained from patients exposed to CFZ and/or BDQ occurred within the *mmpR* gene (71–80%) (24, 40, 46, 47). Taken together, the abovementioned results suggest that *mmpR* mutations play a major role in BDQ and CFZ resistance. Intriguingly, most MmpR mutant amino acid substitutions associated with drug resistance in this study (79%, 11/14) were located within the MmpR DNA-binding domain (residues 32–97), thus indicating this domain is a potential hotspot where drug resistance-induced amino acid changes with low fitness costs are frequently found. Similarly, results described in a recent systematic review of mutations associated with BDQ and CFZ resistance revealed that amino acid sequence changes resulting from BDQ/CFZ-induced *mmpR* mutations occurred most frequently within three MmpR DNA-binding domain regions (residues 64–66, 46–48, and 70–72) (48).

The most important limitation of the present study pertains to the fact that some mutations induced by *in vitro* drug treatments of our clinical isolates may have remained undetected. Nevertheless, parent strains studied herein were derived from clinical isolates and thus were more closely related to clinically relevant organisms than standard laboratory strains. However, previously reported Q76*, G78A, and F93S MmpR mutants (25, 44, 47), and mutants reported here for the first time (to our knowledge), should be verified for clinical relevance through analysis of additional drug-resistant clinical MTB isolates. Furthermore, it has been suggested that the development of BDQ resistance *in vitro* is a dynamic process, such that *mmpR* mutation-induced acquisition of low-level BDQ resistance may be an initial transient, but required, step for subsequent acquisition of high-level BDQ resistance through mutation of *atpE* (49). Nonetheless, we did not engage in multiple exposure-based screening processes that might have enabled us to observe the abovementioned dynamic phenomenon. Moreover, the MIC breakpoint used here to classify MTB strains as BDQ resistant has not been independently validated, while WHO-endorsed BDQ MIC breakpoints that were chosen to minimize false-positive resistance results may lead to missed detection of BDQ-resistant TB cases associated with only small BDQ MIC increases. Finally, we did not conduct whole-genome sequencing (WGS) of BDQ/CFZ-resistant progeny strains that lacked detected mutations, thus warranting additional WGS-based studies to explore additional novel drug resistance-inducing MTB gene mutations.

## Data Availability

The *mmpR* and *pepQ* nucleotide sequences of all mutant strains were deposited in GenBank with accession numbers OR188353 to OR188386.

## SUPPLEMENTAL MATERIAL

Supplemental tables spectrum.00090-23-s0001.docx Tables S1 to S2.

ASM does not own the copyrights to Supplemental Material that may be linked to, or accessed through, an article. The authors have granted ASM a non-exclusive, world-wide license to publish the Supplemental Material files. Please contact the corresponding author directly for reuse.
