# Supplementary material for: Spontaneous mutational patterns and novel mutations for bedaquiline and clofazimine resistance in Mycobacterium tuberculosis
Source: Microbiol Spectr. 2023 Aug 30;11(5):e00090-23. doi: 10.1128/spectrum.00090-23 (PMC10581187; doi:10.1128/spectrum.00090-23)
Supplement: Supplemental tables — Tables S1 to S2. [file spectrum.00090-23-s0001.docx]

**Supplementary table S1. Detail information for all parental strains.**

| Testing Batch | Strain ID | Spoligotyping | MIC (µg/ml) | |
| --- | --- | --- | --- | --- |
|  |  |  | BDQ | CFZ |
| 1 | H37Rv-1 | - | 0.03 | 0.25 |
| 1 | DS-1 | Beijing | 0.03 | 0.25 |
| 1 | DS-2 | Beijing | 0.06 | 0.25 |
| 1 | DS-3 | T1 | 0.03 | 0.25 |
| 1 | DS-4 | Beijing | 0.125 | 0.5 |
| 1 | DS-5 | Beijing | 0.03 | 0.5 |
| 1 | DS-6 | Beijing | 0.06 | 0.5 |
| 1 | DS-7 | Beijing | 0.03 | 0.5 |
| 1 | DS-8 | Beijing | 0.03 | 0.5 |
| 1 | DS-9 | T1 | ≤0.016 | 0.25 |
| 1 | DS-10 | T1 | 0.06 | 0.25 |
| 1 | DS-11 | Beijing | 0.125 | 2 |
| 1 | DS-12 | Undefined^a^ | 0.25 | 1 |
| 1 | DS-13 | Beijing | 0.5 | 1 |
| 1 | DS-14 | Beijing | 0.06 | 2 |
| 1 | DS-15 | Beijing | 0.125 | 2 |
| 2 | H37Rv-2 | - | 0.03 | 0.5 |
| 2 | MDR-1 | Beijing | ≤0.016 | 0.5 |
| 2 | MDR-2 | Beijing | 0.03 | 0.25 |
| 2 | MDR-3 | Beijing | ≤0.016 | 0.25 |
| 2 | MDR-4 | Beijing | 0.03 | 0.5 |
| 2 | MDR-5 | Beijing | 0.03 | 0.5 |
| 2 | MDR-6 | Beijing | ≤0.016 | 0.125 |
| 2 | MDR-7 | Beijing | 0.06 | 0.25 |
| 2 | MDR-8 | Beijing | 0.03 | 0.5 |
| 2 | MDR-9 | Beijing | 0.03 | 0.5 |
| 2 | MDR-10 | Beijing | 0.06 | 0.5 |
| 2 | MDR-11 | Beijing | 0.25 | 1 |
| 2 | MDR-12 | Beijing | 0.5 | 0.5 |
| 2 | MDR-13 | Beijing | 0.25 | 2 |
| 2 | MDR-14 | Beijing | 0.25 | 2 |
| 2 | MDR-15 | Beijing | 0.125 | 2 |
| 3 | H37Rv-3 | - | 0.03 | 0.25 |
| 3 | XDR-1 | Beijing | 0.03 | 0.125 |
| 3 | XDR-2 | Beijing | 0.03 | 0.5 |
| 3 | XDR-3 | Beijing | ≤0.016 | 0.5 |
| 3 | XDR-4 | Beijing | 0.06 | 0.25 |
| 3 | XDR-5 | Beijing | 0.06 | 0.5 |
| 3 | XDR-6 | Beijing | 0.06 | 0.5 |
| 3 | XDR-7 | Beijing | 0.06 | 0.25 |
| 3 | XDR-8 | Beijing | 0.03 | 0.25 |
| 3 | XDR-9 | Beijing | 0.06 | 0.25 |
| 3 | XDR-10 | Beijing | 0.06 | 0.25 |
| 3 | XDR-11 | Beijing | 0.125 | 2 |
| 3 | XDR-12 | Beijing | 0.06 | 1 |
| 3 | XDR-13 | Beijing | 0.25 | 1 |
| 3 | XDR-14 | Beijing | 0.5 | 4 |
| 3 | XDR-15 | Beijing | 0.25 | 2 |

^a^ "Undefined" means that the type was not found in the SITVIT2 database.

**Supplementary table S2. Detail information for all progeny strains.**

| Testing Batch | Strain ID | Parental strain | Drug/concentration | Gene | Nucleotide change | Amino acid change | MIC (µg/ml) | |
| --- | --- | --- | --- | --- | --- | --- | --- | --- |
|  |  |  | (μg/mL) |  |  |  | BDQ | CFZ |
| 1 | H37Rv-1 | - | - | - | - | - | 0.03 | 0.25 |
| 1 | 2-6 | DS-1 | BDQ/0.125 | *mmpR* | 70G>T | G24C | 0.25 | 2 |
| 1 | 2-24 | DS-3 | BDQ/0.125 | *mmpR* | 134T>G | V45G | 0.125 | 4 |
| 1 | 2-37^a^ | DS-6 | BDQ/0.125 | *mmpR* | 194G>A | G65E | 0.25 | 2 |
| 1 | 2-52^b^ | DS-10 | BDQ/0.125 | *mmpR* | 233G>C | G78A | 0.25 | 2 |
| 1 | 2-53^b^ | DS-10 | BDQ/0.125 | *mmpR* | 233G>C | G78A | 0.25 | 2 |
| 1 | 2-5 | DS-1 | BDQ/0.125 | *mmpR* | 253G>T | V85P | 0.125 | 2 |
| 1 | 2-54^c^ | DS-10 | BDQ/0.125 | *mmpR* | 466C>T | R156*^i^ | 0.25 | 2 |
| 1 | 2-44 | DS-8 | CFZ/0.5 | *mmpR* | 116T>C | L39S | 0.06 | 1 |
| 1 | 2-25 | DS-4 | CFZ/0.5 | *mmpR* | 119T>G | L40W | 0.5 | 4 |
| 1 | 2-33 | DS-6 | CFZ/0.5 | *mmpR* | 124T>C | W42R | 0.25 | 4 |
| 1 | 2-20^d^ | DS-3 | CFZ/0.5 | *mmpR* | 134T>G | V45G | 0.125 | 4 |
| 1 | 2-21^d^ | DS-3 | CFZ/1 | *mmpR* | 134T>G | V45G | 0.125 | 4 |
| 1 | 2-34 | DS-6 | CFZ/1 | *mmpR* | 151C>T | Q51* | 0.125 | 2 |
| 1 | 2-47 | DS-9 | CFZ/0.5 | *mmpR* | 257C>T | A86V | 0.125 | 2 |
| 1 | 2-26 | DS-4 | CFZ/1 | *mmpR* | 278T>C | F93S | 0.5 | 4 |
| 1 | 2-48 | DS-9 | CFZ/0.5 | *mmpR* | 302C>A | A101E | 0.03 | 1 |
| 1 | 2-43 | DS-8 | CFZ/0.5 | *mmpR* | 425T>C | L142P | 0.125 | 2 |
| 1 | 2-28 | DS-5 | CFZ/0.5 | *mmpR* | 435_436insGCGGATTTCACAAAGCAG^g^ | Y145_M146insADFTKQ | 0.125 | 1 |
| 1 | 2-19 | DS-2 | CFZ/1 | *pepQ* | 947T>G | L316R | 0.25 | 2 |
| 1 | 2-18 | DS-2 | CFZ/0.5 | *pepQ* | 1060delA^h^ | T354fs^j^ | 0.5 | 2 |
| 2 | H37Rv-2 | - | - | - | - | - | 0.03 | 0.25 |
| 2 | 2-96^e^ | MDR-7 | BDQ/0.125 | *pepQ* | 735G>A | W245* | 0.25 | 4 |
| 2 | 2-103 | MDR-9 | CFZ/0.5 | *mmpR* | 109G>C | G37R | 0.25 | 1 |
| 2 | 2-58^a^ | MDR-1 | CFZ/0.5 | *mmpR* | 194G>A | G65E | 0.25 | 2 |
| 2 | 2-92 | MDR-7 | CFZ/1 | *mmpR* | 198delG | G66fs | 0.5 | 4 |
| 2 | 2-79 | MDR-4 | CFZ/0.5 | *mmpR* | 226C>T | Q76* | 0.125 | 1 |
| 2 | 2-98 | MDR-8 | CFZ/1 | *mmpR* | 248T>C | L83P | 0.5 | 2 |
| 2 | 2-97 | MDR-8 | CFZ/0.5 | *mmpR* | 436delA,437delT | M146fs | 0.25 | 2 |
| 2 | 2-91^e^ | MDR-7 | CFZ/0.5 | *pepQ* | 735G>A | W245* | 0.125 | 2 |
| 3 | H37Rv-3 | - | - | - | - | - | 0.03 | 0.25 |
| 3 | 1-15^f^ | XDR-1 | BDQ/0.125 | *mmpR* | 32delG | G11fs | 0.125 | 2 |
| 3 | 1-16^f^ | XDR-1 | BDQ/0.125 | *mmpR* | 32delG | G11fs | 0.125 | 2 |
| 3 | 1-77^c^ | XDR-7 | BDQ/0.125 | *mmpR* | 466C>T | R156* | 0.25 | 2 |
| 3 | 1-78 | XDR-9 | BDQ/0.125 | *pepQ* | 347_348insG | D116fs | 0.25 | 4 |
| 3 | 1-76^f^ | XDR-1 | CFZ/0.5 | *mmpR* | 32delG | G11fs | 0.125 | 2 |
| 3 | 1-75 | XDR-10 | CFZ/0.5 | *mmpR* | 412_413insG | E138fs | 0.5 | 4 |

^a^ 2-37 and 2-58 have the same mutation in *mmpR* 194G>A.

^b^ 2-52 and 2-53 have the same mutation in *mmpR* 233G>C.

^c^ 2-54 and 1-77 have the same mutation in *mmpR* 466C>T.

^d^ 2-20 and 2-21 have the same mutation in *mmpR* 134T>G.

^e^ 2-96 and 2-91 have the same mutation in *mmpR* 735G>A.

^f^ 1-15, 1-16 and 1-76 have the same mutation in *mmpR* 32delG.

^g^ ins: insertion.

^h^ del: deletion.

^i^*: stop codon.

^j^ fs: frame shift.
